# Harnessing Lignocellulosic Crops for Phytomanagement of Contaminated Soils: A Multi-Country Study

**DOI:** 10.3390/plants13192671

**Published:** 2024-09-24

**Authors:** Giorgio Testa, Barbara Rachele Ciaramella, Ana Luisa Fernando, Danai Kotoula, Danilo Scordia, Leandro Augusto Gomes, Salvatore Luciano Cosentino, Efthymia Alexopoulou, Eleni G. Papazoglou

**Affiliations:** 1 Dipartimento di Agricoltura, Alimentazione e Ambiente (Di3A), University of Catania, Via Santa Sofia 100, 95123 Catania, Italy; barbara.ciaramella@phd.unict.it (B.R.C.); cosentin@unict.it (S.L.C.); 2 MEtRICs, CubicB, Chemistry Department (DQ), NOVA School of Science and Technology|NOVA FCT, Universidade Nova de Lisboa, Campus de Caparica, 2829-516 Caparica, Portugal; ala@fct.unl.pt; 3 Department of Crop Science, Agricultural University of Athens, 11855 Athens, Greece; danai.env@gmail.com; 4 Dipartimento di Scienze Veterinarie, University of Messina, Via G. Palatucci s.n., 98168 Messina, Italy; danilo.scordia@unime.it; 5 CNR-ITAE, Salita Santa Lucia Sopra Contesse 5, 98126 Messina, Italy; leandrogomesfsc@gmail.com; 6 Center for Renewable Energy Sources, Biomass Department, 19009 Pikermi Attiki, Greece; ealex@cres.gr

**Keywords:** phytoremediation, heavy metals, contaminated soil, biomass, switchgrass, sorghum, giant reed, African fodder cane, miscanthus, cadmium, lead, nickel, zinc

## Abstract

The dwindling availability of agricultural land, caused by factors such as rapid population growth, urban expansion, and soil contamination, has significantly increased the pressure on food production. To address this challenge, cultivating non-food crops on contaminated land has emerged as a promising solution. This approach not only frees up fertile soil for food production but also mitigates human exposure to contaminants. This work aimed to examine the impact of soil contamination with Cd, Pb, Ni, and Zn on the growth, productivity, metal accumulation, and the tolerance of five lignocellulosic non-food crops: switchgrass (*Panicum virgatum* L.), biomass sorghum (*Sorghum bicolor* L. Moench), giant reed (*Arundo donax* L.), African fodder cane (*Saccharum spontaneum* L. spp. *aegyptiacum* Willd. Hackel), and miscanthus (*Miscanthus* × *giganteus* Greef et Deu.). A two-year pot experiment was conducted in Greece, Italy, and Portugal, following the same protocols and applying various levels of metals: Cd (0, 4, 8 mg kg^−1^), Pb and Zn (0, 450, 900 mg kg^−1^), and Ni (0, 110, 220 mg kg^−1^). The experimental design was completely randomized, with three replicates for each treatment. The results showed that switchgrass and sorghum generally maintained their height and productivity under Cd and Pb stress but were adversely affected by high Zn and Ni concentrations. Giant reed and African fodder cane showed reduced height and productivity at higher Ni and Zn levels. Miscanthus exhibited resilience in height but experienced productivity reductions only at the highest Zn concentration. Heavy metal uptake varied among crops, with switchgrass and sorghum showing high Cd and Pb uptake, while giant reed accumulated the most Cd and Zn. Miscanthus had the highest Ni accumulation. The tolerance indices indicated that switchgrass and sorghum were more tolerant to Cd and Zn at lower concentrations, whereas miscanthus had lower tolerance to Cd but a higher tolerance to Zn at higher concentrations. Giant reed and African fodder cane demonstrated stable tolerance across most heavy metals. Accumulation indices highlighted the effectiveness of switchgrass and sorghum in Cd and Pb uptake, while miscanthus excelled in Ni and Zn accumulation. The cluster analysis revealed similar responses to heavy metal stress between African fodder cane and giant reed, as well as between sorghum and miscanthus, with switchgrass displaying distinct behavior. Overall, the study highlights the differential tolerance and accumulation capacities of these crops, indicating the potential for phytoremediation applications and biomass production in heavy metal-contaminated soils.

## 1. Introduction

Agricultural land is a natural resource and one of the most important indicators of economic growth. However, in the last decades, available agricultural land has declined due to rapid population growth, land-use changes, abandonment, degradation, desertification, and soil contamination [1,2,3]. Furthermore, the rising demand for food necessitates increased productivity within the already limited agricultural land, exacerbating conflicts over land-use changes and restricting the availability for non-food purposes [4,5]. However, the cultivation, processing, and utilization of non-food crops remain indispensable because they (i) support bioenergy and biofuel production, enhancing energy security and allowing rapid responses to fluctuations in demand, and serve as a source for various fuels and energy conversion techniques [6]; (ii) they supply many industries with biobased polymers, nanocellulose, building materials [7], biolubricants, and biosurfactants, to name a few [8,9,10], thus impacting the energy, transport, and industry sectors [11,12], and (iii) they offer environmental advantages by contributing to the reduction of greenhouse gases, helping combat climate change, and providing social benefits, especially in rural areas.

Therefore, how can non-food crops be cultivated to minimize impacts on land-use conflicts arising from competition for food and feed? One promising approach is to utilize contaminated land for cultivating non-food crops, releasing valuable fertile and healthy soils for food production [13]. Simultaneously, this strategy helps mitigate human exposure to contaminants. In addition, these lands are unsuitable for the cultivation of food crops due to the stressful conditions caused by contamination, which also undermine the site’s economic viability. Numerous studies have demonstrated that high levels of toxic elements, such as Cd, Pb or Ni, in the soil can adversely affect seed germination and plant growth by reducing photosynthetic activities, nutrient uptake, and water nutrition, among other detrimental effects [14,15]. Additionally, soil contamination can lead to several environmental problems, including desertification, water resource contamination, transport of contaminants to nearby healthy soils via air and runoff, propagation of contaminants along the food chain, and loss of ecosystem services [16].

An eco-friendly solution for decontamination is phytoremediation, a technology that employs plants to remove pollutants from the environment or render them harmless by stabilization [17]. The development of this technology is being driven primarily by the high cost of many other soil remediation methods, as well as by the desire to use a ‘green’, sustainable process [18,19,20]. Phytomanagement is defined as using plants to reduce and control the risks of soil contamination, while making profitable and sustainable use of this resource by producing marketable biomass [21,22]. The development of phytomanagement aligns with the perspective that many contaminated soils can still be valuable resources if managed sustainably. Therefore, selecting suitable crops to be used in this process is crucial for effectively remediating contaminated land and creating an economically viable value chain. The selected crops should be able to tolerate contamination while maintaining comparable productivity and quality [23]. Considering these characteristics, industrial crops present a viable option due to their resilience to stressful conditions and their ability to produce biomass for various applications. However, it is important to note that yields and biomass quality can still be impacted by the degree of contamination [24]. In addition, cultivating non-food crops on contaminated soils and producing biomass may provide supplementary income opportunities for farmers [25].

Heavy metals are particularly noteworthy contaminants that can render a site unsuitable for food crops [26]. Understanding the impact of each heavy metal on non-food crops is crucial to selecting the most suitable crop for the phytomanagement of contaminated soils. In the context of the MAGIC project, funded by the European Commission under the H2020 Program (https://magic-h2020.eu/, accessed on 25 July 2024), which aimed to assist farmers in choosing suitable industrial crops for marginal lands, the present study addressed the effects of different heavy metals on the yield and phytoremediation potential of non-food industrial crops. In this work, five lignocellulosic crops, namely switchgrass (*Panicum virgatum* L.), biomass sorghum (*Sorghum bicolor* L. Moench), giant reed (*Arundo donax* L.), African fodder cane (*Saccharum spontaneum* L. spp. *aegyptiacum* Willd. Hackel), and miscanthus (*Miscanthus* × *giganteus* Greef et Deu.) were selected to be tested in soils contaminated with cadmium (Cd), lead (Pb), nickel (Ni), and zinc (Zn). These heavy metals are commonly found in soil as a result of various urban, industrial, and agricultural activities that lead to contamination. The five lignocellulosic crops were chosen for their ability to grow in harsh conditions, including soils contaminated with heavy metals, and for their potential to produce high yields of biomass, suitable for multiple industrial applications [24].

Some studies have been carried out on the proposed crops, with a low content of heavy metals in the soil. For example, giant reed has shown a notable capacity to withstand contamination by heavy metals. In pot experiments, the plant demonstrated resilience to various metals, although yields were affected by higher concentrations. In particular, the aboveground yields were diminished by 30–70% in soils contaminated with certain metals, except for zinc and nickel. The belowground biomass remained largely unaffected, indicating that while heavy metals can impact its growth, giant reed is capable of thriving in contaminated environments. Furthermore, giant reed has been identified as a promising candidate for phytoremediation, effectively absorbing and concentrating heavy metals from the soil [27]. This capacity enables the decontamination of polluted regions while simultaneously providing biomass for energy production [17,22]. Furthermore, switchgrass displays a notable resilience to heavy metals. In analogous studies, the plant demonstrated the capacity to maintain yields in the presence of several metals, including zinc, lead, copper, and cadmium. However, higher concentrations of nickel resulted in a notable decline in yield. The plant’s belowground biomass demonstrated notable resilience with regard to the impact on overall productivity. Although there are not many studies on sorghum and miscanthus in the context of heavy metal contamination, these crops are generally recognized for their adaptability and potential for bioenergy production. In particular, sorghum is renowned for its drought resistance and capacity to flourish in suboptimal soil conditions, which may extend to its tolerance to heavy metals. *Sorghum bicolor* is primarily grown for industrial purposes, particularly for bioenergy production, but its varieties have different applications. Dual-purpose sorghum can be grown for both grain and forage, while forage sorghum is used exclusively for animal feed. Sweet sorghum has traditionally been cultivated for syrup or molasses production. In recent years, biomass sorghum has emerged as a promising feedstock for second-generation (2G) biofuel production due to its high biomass yield and adaptability to diverse agroecological conditions, including marginal lands. Studies have highlighted that sorghum requires fewer agronomic inputs, such as irrigation and fertilizers, compared to other crops, making it a more sustainable choice. It also demonstrates superior drought tolerance, with high water use efficiency (WUE) and radiation use efficiency (RUE), which makes it an ideal candidate for bioenergy in regions with limited water resources [28,29,30]. Moreover, the residues from sorghum, such as bagasse and straw, are well-suited for 2G biofuel production, similarly to sugarcane [31,32]. As such, high-biomass sorghum offers a sustainable option for bioenergy without competing with food crops for fertile land or water resources.

Miscanthus, a species frequently utilized for bioenergy purposes, exhibits comparable characteristics; however, further investigation is necessary to fully comprehend its response to heavy metal stress. *S. spontaneum* has been observed to exhibit a notable capacity for the accumulation of heavy metals, with a particular affinity for lead (Pb). One study examined the effects of varying Pb concentrations (0, 100, 200, 300 ppm) on *S. spontaneum* over an eight-week period. The findings revealed that with elevated concentrations of Pb, the roots of *S. spontaneum* exhibited heightened accumulation, indicating a potential for increased detoxification processes. Additionally, analysis of the phytochelatin synthase (PCS) gene expression, linked to metal detoxification, showed elevated activity in the roots, suggesting an active response to heavy metal stress [33,34].

## 2. Results

### 2.1. Height of the Plants

**Figure 1:**
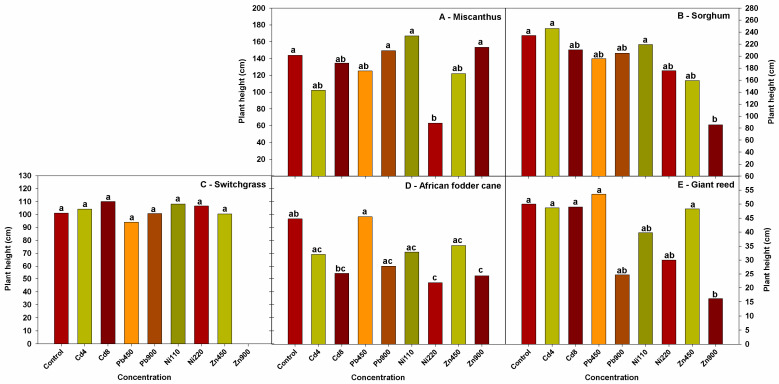
Plant height (cm) of the five lignocellulosic crops ((**A**): miscanthus, (**B**) sorghum, (**C**) switchgrass, (**D**) African fodder cane (**E**), giant reed) subjected to different Cd, Ni, Pb, and Zn concentrations (mean values of years 2019 and 2020, *n* = 3). Different lower-case letters indicate significantly different means (*p* < 0.05).

When comparing the five crops tested under the various treatments applied to the soil, Figure 1 shows that switchgrass, sorghum, giant reed, and miscanthus did not exhibit a significant reduction in height compared to the control in Cd- and Pb-contaminated soil. However, African fodder cane showed a significant reduction in height at the highest concentrations of Cd and Zn in the soil.

In Ni-contaminated soil, African fodder cane and miscanthus were significantly affected in terms of height by the presence of the highest concentration of this heavy metal. Giant reed and sorghum showed a slight reduction in plant height at the Ni 220 mg kg^−1^ concentration, while no significant reduction was observed in switchgrass.

Different results were observed in Zn-contaminated soil. At the highest concentration, switchgrass seeds did not germinate, while sorghum, giant reed, and African fodder cane showed a significant reduction in plant height. In contrast, miscanthus did not show any reduction in height.

### 2.2. Plant Productivity in Response to Heavy Metals

In the two-way ANOVA, both applied levels of heavy metals and the tested crops showed significant differences, while in the interaction between the tested crops and the applied levels of heavy metals, a significant difference was observed only in the Zn-contaminated soil (Table 1).

Across the average of two production years, there was no difference in the plant dry weight of miscanthus and African fodder cane between the control and the different levels of heavy metals applied. A similar result was observed for the productivity of switchgrass, but at Zn900 all plants died in both years (Table 2). On the other hand, giant reed showed a significant reduction in yield at the highest concentration of Zn (Zn 900 mg kg^−1^), Pb (Pb 900 mg kg^−1^), and Ni (Ni 220 mg kg^−1^). For sorghum, a significant reduction was observed for Zn 900 mg kg^−1^, while the aboveground yield was higher for Ni 110 mg kg^−1^ compared to the control.

### 2.3. Heavy Metals Concentration and Accumulation

Regarding the concentration of heavy metals in the aboveground parts, the five crops exhibited similar behavior across the contamination levels. In Cd-contaminated soil, the highest concentration of heavy metals was found in giant reed, followed by African fodder cane, sorghum, and switchgrass (Table 3). Although the concentration in miscanthus was lower compared to the other crops, the amount of metal concentrated in Cd 8 mg kg^−1^ treatment was more than three times higher than in Cd 4 mg kg^−1^ treatment, a difference that was not observed in the other crops.

In Pb-contaminated soils, sorghum exhibited the highest concentration of heavy metals, followed by switchgrass, giant reed, miscanthus, and African fodder cane. In sorghum, the difference in concentration between the two levels of Pb was more than double, while miscanthus showed no difference in Pb concentration between the Pb 450 and Pb 900 mg kg^−1^ level.

In Ni-contaminated soil, the highest concentration of heavy metals was observed in miscanthus, followed by switchgrass, giant reed, and African fodder cane. Sorghum had the lowest concentration, and miscanthus showed a more than two-fold increase in concentration between the two different levels of Ni contamination.

In Zn-contaminated soil, the highest concentration of heavy metals was found in giant reed, followed by African fodder cane, miscanthus, and sorghum. Switchgrass did not survive at the highest Zn concentration. Comparing the two levels of Zn in the soil, giant reed accumulated three times more at the Zn-high level than at the Zn-low level, while miscanthus accumulated nearly twice as much.

**Figure 2:**
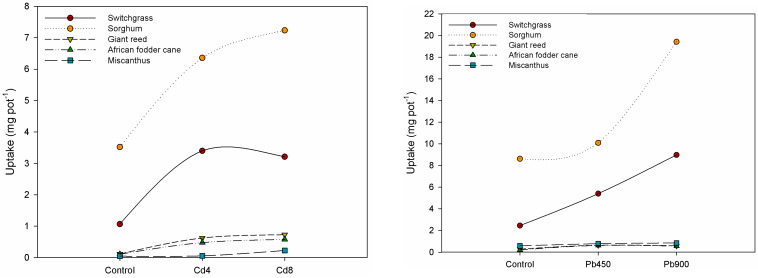

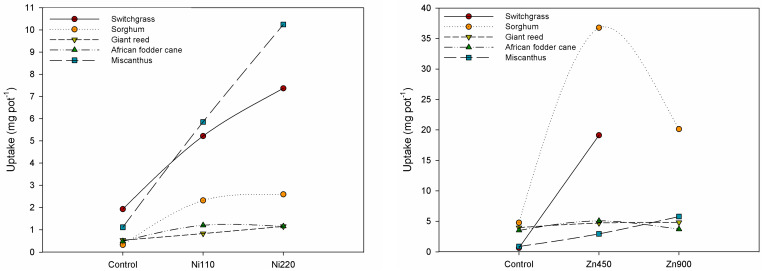
Heavy metal uptake (mg pot^−1^) in the aerial biomass of the crops.

The uptake of heavy metals by the five crops was calculated based on the total aboveground biomass and the corresponding metal concentrations (Figure 2).

Cadmium uptake was higher in sorghum and switchgrass. However, as the Cd concentration increased, sorghum’s uptake decreased slightly due to reduced productivity.

A similar trend was observed in miscanthus, giant reed, and African fodder cane. The lead uptake was also higher in sorghum and switchgrass, with the uptake increasing as the Pb concentration rose. Similar behavior was also observed in miscanthus, giant reed, and African fodder cane.

Zinc uptake was higher in sorghum and switchgrass. However, at the Zn 900 mg kg^−1^ concentration, the uptake decreased in sorghum, while the switchgrass plants did not survive at this level. A similar decline in uptake was observed in the giant reed and African fodder cane. In contrast, miscanthus showed a slight increase in Zn uptake with higher metal concentrations in the soil.

Finally, in the Ni-contaminated soil, all crops exhibited increased uptake as the metal concentration in the soil rose. The highest concentration was observed in miscanthus, followed by switchgrass, sorghum, giant reed, and African fodder cane.

### 2.4. Phytoremediation Index

#### 2.4.1. Tolerance Index

**Figure 3:**
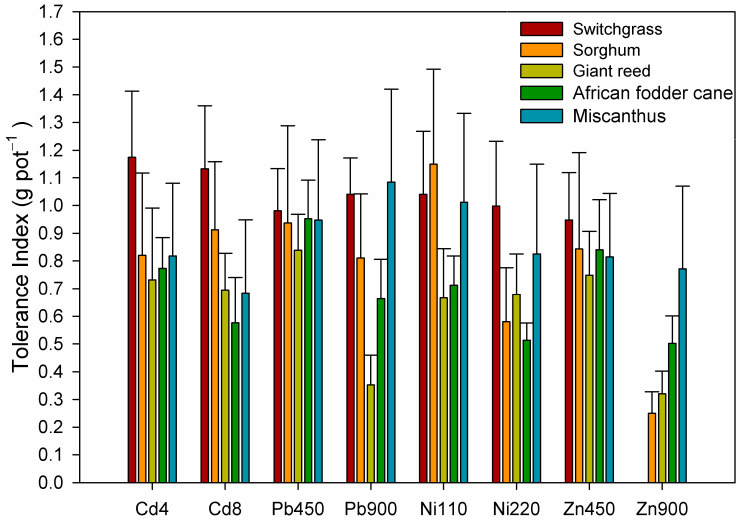
Tolerance index of the five crops affected by heavy metal treatments. Bars represent standard deviation.

The tolerance index, which compares the aboveground biomass produced in the control pots to the aboveground biomass produced in the contaminated pots, helps us to better understand which of the five tested crops was more tolerant to the presence of each heavy metal in the soil. Among all the heavy metals tested, Figure 3 shows that switchgrass, followed by sorghum, were likely more tolerant to Cd stress, even at the highest concentration. In contrast, miscanthus grown in Cd-polluted soils showed the lowest tolerance.

In Pb-contaminated soils, all the crops tested appeared to tolerate the lowest concentration of Pb. Switchgrass and miscanthus had the highest tolerance index, while at the highest Pb concentration in the soil, giant reed’s tolerance index value was halved.

In Ni-contaminated soils, the tolerance index decreased for sorghum and miscanthus, and slightly for switchgrass, as the heavy metal concentration in the soil increased. For African fodder cane and giant reed, the tolerance index remained similar between the two concentrations. The latter showed the lowest tolerance index in Ni-contaminated soils, while the highest values were observed for sorghum and switchgrass at Ni 110 and Ni 220 mg kg^−1^, respectively.

In Zn-contaminated soils, the tolerance of all five crops decreased as the concentration in the soil increased. Switchgrass showed the highest tolerance index at Zn 450 mg kg^−1^, but the plants did not germinate at Zn 900 mg kg^−1^. The highest tolerance to Zn 900 mg kg^−1^ was observed in miscanthus, while all other crops showed a tolerance index lower than 0.50.

#### 2.4.2. Accumulation Index

The accumulation index indicates the amount of heavy metal that the plants can take up compared to the control.

**Figure 4:**
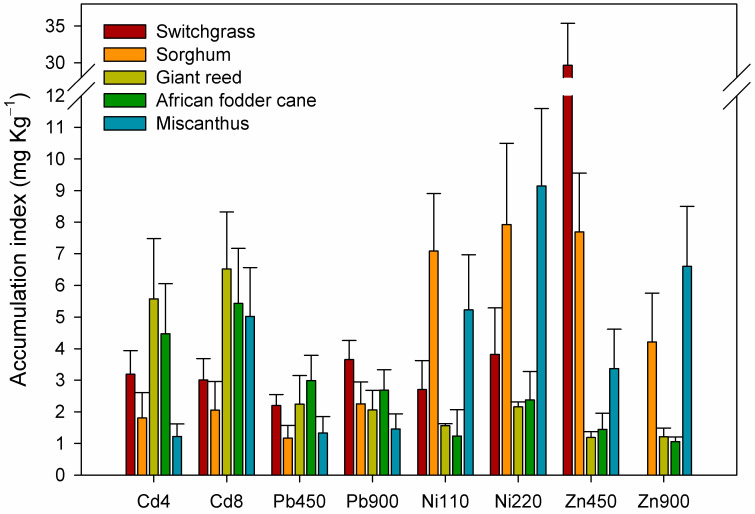
Accumulation index of the five crops affected by the heavy metal treatments. Bars represent standard deviation.

In Cd-contaminated soil, the highest accumulation index was observed in giant reed, followed by African fodder cane (Figure 4). With increasing Cd concentration in the soil, miscanthus increased the accumulation index seven-fold.

In Pb-contaminated soil, although the highest accumulation index was observed in African fodder cane at the lowest concentration, at Pb 900 mg kg^−1^ the highest accumulation index was recorded in switchgrass.

In Ni-contaminated soils, important results were observed for sorghum and miscanthus. With increasing Ni concentration in the soil, the accumulation index in these two crops increased, indicating their ability to phytoextract high amounts of this metal.

The highest accumulation index was observed in switchgrass at Zn 450 mg kg^−1^, followed by sorghum and miscanthus, while at Zn 900 mg kg^−1^, the highest accumulation index was observed in sorghum, followed by miscanthus. As the Zn concentration in the soil increased, only miscanthus was able to increase the amount of the metal accumulated in the plants.

### 2.5. Principal Component and Cluster Analysis

**Figure 5:**
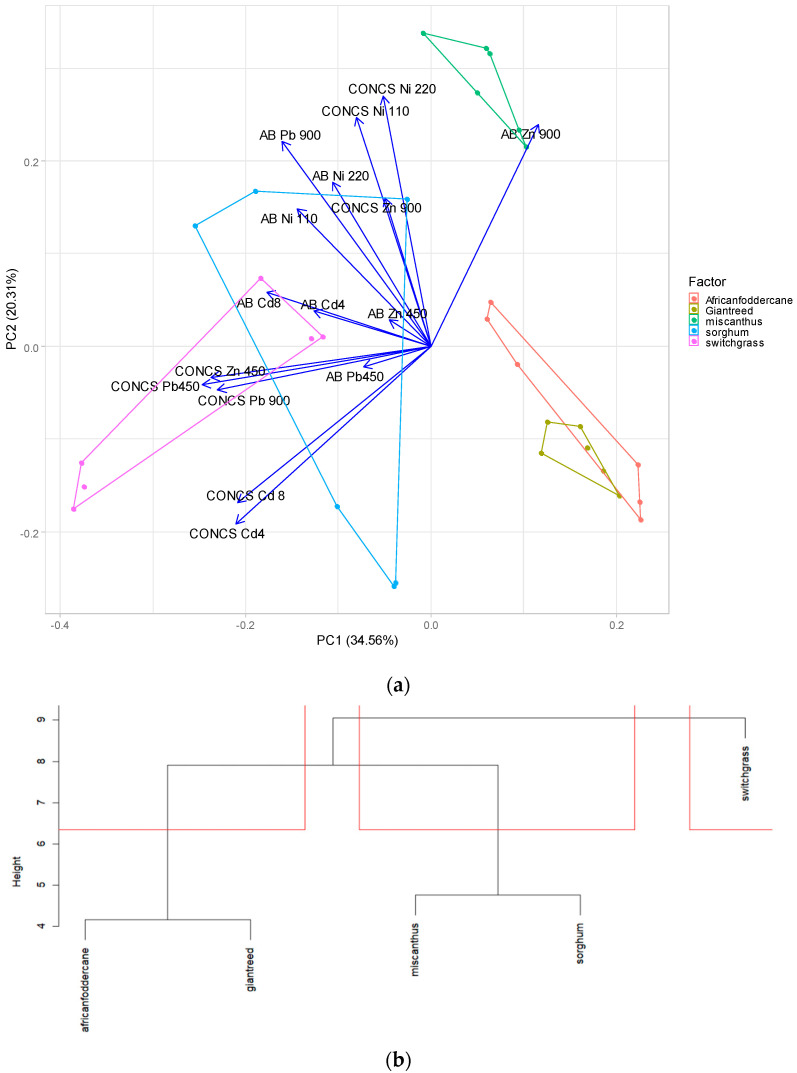
Principal component analysis (**a**) and cluster analysis (**b**) of main factor (crops species) and the variable (yield and concentration of heavy metal).

The cluster analysis was performed to better understand how the tested species could be compared to the different heavy metal concentrations in the soil. For this dendrogram, the heavy metal concentrations in the plant biomass and the yields were used as variables, while the five crops were used as the factors. Figure 5 illustrates that, despite some different behavior, African fodder cane showed a similar result to that of giant reed compared to the other crops. Miscanthus and sorghum also demonstrated similar behavior in terms of productivity and aboveground biomass concentration. Finally, switchgrass differed from all the other crops.

## 3. Discussion

In the Mediterranean environment, it is important to promote crops that (i) have high yield potential, (ii) adapt well to current and future environmental constraints, and (iii) are capable of growing under water deficit conditions and with reduced inputs [35]. Belonging to the Poaceae family, switchgrass, sorghum, giant reed, African fodder cane, and miscanthus are lignocellulosic biomass grasses that have been successfully grown on marginal or contaminated lands. These crops demonstrate considerable environmental benefits, including greenhouse gas savings, soil erosion mitigation, improved soil fertility, and enhanced biodiversity [36].

The abiotic stress tolerance that characterizes these crops allows them to be grown in soils with various stress conditions [37], including heavy metal contamination in different concentrations and bioavailability [38].

Considering their mechanisms to resist, tolerate, grow, and remediate toxic metalliferous soils, studying these plants as potential pioneer species for the phytoremediation of heavy metal-contaminated soils is of significant interest [39].

Moreover, these plants offer various advantages such as fast growth, wide distribution, high yields, and being non-food energy crops [40]. Furthermore, their biomass can be used to produce advanced biofuels or bio-based products due to their high productivity and cellulose/hemicellulose composition [25]. The results presented in this study contribute to a better understanding of the behavior of these five lignocellulosic crops when grown in soils contaminated with Cd, Pb, Zn, and Ni at two different levels.

Cadmium is a non-essential element that is detrimental to plant growth and development. It is recognized as a significant pollutant due to its high toxicity and high solubility in water. Cadmium can also disrupt the uptake of minerals by plants, affecting the availability of nutrients in the soil. Exposure to cadmium in nutrient solutions has been reported to impact stomatal opening, transpiration, and photosynthesis [38]. Although Cd ions are primarily retained in the roots, their uptake and transport are influenced by the soil pH and dissolved organic carbon [40,41]. The accumulation of metals in plants depends on the uptake capacity and the availability of intracellular binding sites, with the concentration and affinities of chelating molecules playing a crucial role [38]. Additionally, the presence and selectivity of transport mechanisms affect metal accumulation rates [39]. In this study, Cd impacted the height and productivity of African fodder cane and miscanthus, especially at the highest concentration of contamination. Despite the adverse effects of this heavy metal, the concentration of Cd in the aerial biomass of both African fodder cane and miscanthus increased significantly compared to the control.

Xia et al. [42] also studied the growth and heavy metal accumulation of sugarcane grown in artificially contaminated soil with different concentrations of Cd. They found that sugarcane, with its large biomass, has a high capacity to tolerate and accumulate Cd. Furthermore, plant species such as sugarcane can survive by activating key enzymes like catalase (CAT), superoxide dismutase (SOD), and glutathione reductase (GR) [43].

Zgorelec et al. [44] observed similar results for miscanthus in an experimental pot study conducted over three years. Compared to the first year, they found a decrease in the yield of *Miscanthus* × *giganteus* in the second and third years, both in the control treatment and in the contaminated soil, ranging from 37% to 55% [44]. Similarly, Ofori-Agyemang et al. 2024 [45] found that high Cd concentrations in soil pore water adversely affected miscanthus roots, resulting in a lower shoot dry weight yield [45].

Different behavior was observed for switchgrass and sorghum, where increasing Cd in the soil did not affect the height or productivity of the plants. Gomes et al. [24] showed that the productivity of switchgrass biomass increased from the first to the second growing cycle when a low concentration of Cd (4 mg kg^−1^) was applied to the soil.

However, Chen et al. [46] used a higher range of Cd concentrations (0–60 mg kg^−1^) and reported that Cd led to a drastic reduction in grass productivity, with losses of up to 63% at the highest contamination level. Similar results were observed for giant reed in Cd-contaminated soil, as reported by Gomes et al. [24].

Lead is one of the most commonly used metals in industrial production due to its softness, high malleability, ductility, low conductivity, and corrosion resistance. However, it tarnishes when exposed to air. Lead does not play an essential role in plant metabolism [47]. The results showed a reduction in productivity, particularly in giant reed and sorghum, when exposed to lead.

Lead contamination in soil was mitigated using giant reed in a 2-year outdoor experiment. The study explored the considerable potential of giant reed for phytoextraction and soil fertility restoration, confirming the ability of this crop to grow in polluted soils [48].

In a study conducted by Osman et al. [49] that tested sorghum with five concentrations of Pb, there was a reduction in the dry weight of roots and shoots in Pb-treated plants. Additionally, Pb significantly impacted the germination and tolerance index across the three cultivars tested.

The feasibility of using edible sugarcane for the rehabilitation of manganese mining sites was investigated, revealing that the concentrations of Cd and Pb in the edible parts of sugarcane were higher than the safety limits [50].

The accumulation of Pb in the biomass increased in all five crops. In a previous phytoremediation study using giant reed cultivated in red mud and a mud–soil mixture (control), the biomass productivity increased over time by 40.4% and 47.2%, respectively, while the concentrations of available Cd, Pb, Co, Ni, and Fe in the soil decreased simultaneously [51]. Soil Pb and Zn fractions were also reduced by giant reed in a 2-year outdoor experiment, which assessed the potential of giant reed for phytoextraction and soil fertility restoration, confirming the crop’s ability to grow on polluted soils. Additionally, the incorporation of compost resulted in the highest biomass production and, consequently, the highest metal uptake by giant reed [48].

Moreover, in a study by Hart et al. [52], switchgrass exhibited low phytotoxicity to Pb. When mixed with specific plant growth regulators and chelating agents, switchgrass showed increased Pb uptake efficiency, indicating its potential for phytoremediation.

Zinc is extensively utilized in various industries, particularly in the automotive, construction, and battery sectors. In plants, Zn serves essential functions as a micronutrient in their metabolism. It affects chlorophyll synthesis and acts as a building block for enzymes, facilitates the metabolism of carbohydrates, proteins, and phosphates, and contributes to RNA formation [47,53]. The Zn requirements vary from plant to plant, but an excess of this heavy metal can be toxic to crops. Despite its essential functions in plant metabolism, excessive Zn can interfere with nutrient uptake, disrupt enzymatic activities, impair photosynthesis, and induce oxidative stress. To mitigate the harmful effects of excess Zn, plants accumulate the metal in the cell walls and vacuoles, both below and above ground. This mechanism enables plants to tolerate high levels of Zn in the soil. A high concentration of Zn inhibits the germination of switchgrass and almost all crops studied have shown reduced productivity, except for miscanthus [54].

The adaptability and phytoremediation capacity of giant reed and miscanthus on contaminated soil were assessed under exposure to concentrations of 450 and 900 mg kg^−1^ dry matter of Zn and Pb, and 300 and 600 mg kg^−1^ dry matter for Cr [54]. The study highlighted the capability of these crops to phytoextract and accumulate heavy metals. In particular, the results confirm that bioaccumulation primarily occurs in the hypogeal part, namely the rhizomes and roots, especially for Pb and Cr, while Zn is more easily transported and accumulated in the aerial fraction.

Although Zn acts as a micro-nutrient in plants, the highest concentration of Zn drastically reduced the productivity of African fodder cane, while giant reed productivity was not affected by the Zn concentration. In giant reed, the increase in soil contamination did not result in a corresponding increase in the concentration of heavy metals in plant tissues, indicating that a limit was reached, which helped to mitigate the toxic effect of Zn at the highest concentrations. In African fodder cane, the accumulation in plant tissues increased with the concentration of Zn in the soil, potentially making this crop more susceptible to the toxic effects. In this study, miscanthus showed an increasing tolerance index with rising Zn levels in the soil, but also a reduction in aerial biomass. As reported by Ofori-Agyemang et al. [45], high concentrations of Zn in the soil negatively affect growth and shoot yields in miscanthus. However, as reported by Pidlisnyuk et al. [21], miscanthus can still increase its tolerance index by storing heavy metals in the roots, which reduces the toxicity of Zn in the soil.

Nickel concentrations in the environment can be accelerated by certain human activities. Examples of anthropogenic Ni deposition in the environment include wastes from fossil fuel power plants, mining, and smelting processes, emissions from the transportation sector, industrial and urban waste, and the steel and cement industries [47,55,56]. In plants, Ni is an essential micronutrient that plays a role in the nitrogen cycle through its presence in some enzymes, such as urease and hydrogenase. The absence of Ni in the soil prevents the plant from completing its growth cycle; however, an excess of Ni can lead to several harmful effects on the plant’s morphological, physiological, and biochemical aspects [56]. In some cereals, excess Ni can be detected by characteristics such as interveinal chlorosis in new leaves, grey-green leaves, and brown-stunted roots [47].

Nickel did not significantly affect the productivity of African fodder cane. Regarding its accumulation potential, an increase was observed in the contaminated pots compared to the control, but the concentration of Ni in the soil did not impact the accumulation potential. A similar accumulation potential was observed for giant reed, where the presence of the contaminant also did not affect the productivity of the crop. However, increasing the concentration of heavy metals in the soil significantly reduced the height of giant reed stems.

The accumulation of Ni in giant reed showed no significant variation in the above-ground biomass from the lowest to the highest concentration of Ni in the soil. This behavior may indicate a limit to the Ni accumulation in this crop. Similarly, a reduction in biomass production was observed in switchgrass and sorghum at Ni220. Al Chami et al. [57], who also tested Pb, Zn, and Ni in *Sorghum bicolor* L., reported that the highest toxicity was associated with Ni contamination. De Bernardi et al. highlighted the phytostabilization potential of sorghum in Ni-contaminated soil, demonstrating its ability to accumulate this heavy metal predominantly in the roots [58].

Gomes et al. [24], in a pot experiment, also tested giant reed and switchgrass using two concentrations of Ni (Ni 110 and Ni 220 mg kg^−1^) over two years. They reported that both species showed reduced productivity at the highest concentration of Ni, but the concentration in the biomass increased with the rising concentration of heavy metal in the soil.

However, differences in biomass production under various abiotic stresses have been observed in these crops by several authors [23]. Therefore, studying the genetic diversity of these species and the differences between varieties/ecotypes could help enhance the heavy metal uptake [48].

Moreover, the selection of energy crops that are well adapted to a specific climatic area, capable of tolerating abiotic stresses, and can grow with reduced agronomic input could be a challenge for further studies.

As more agricultural land becomes contaminated due to various anthropogenic activities, global climate change is predicted to increase the frequency and intensity of droughts in some geographical regions. In arid and semi-arid areas, where evapotranspiration dominates, reduced water availability and longer drought duration are likely to occur [59]. Therefore, it is important to investigate drought-tolerant industrial crops for the Mediterranean region that can grow under heavy metal-contaminated soils in environments with limited water resources. Future work could combine these crops with chelators, microorganisms, or other techniques to remediate polluted soil and improve the extraction of these heavy metals from the soil.

## 4. Materials and Methods

**Figure 6:**
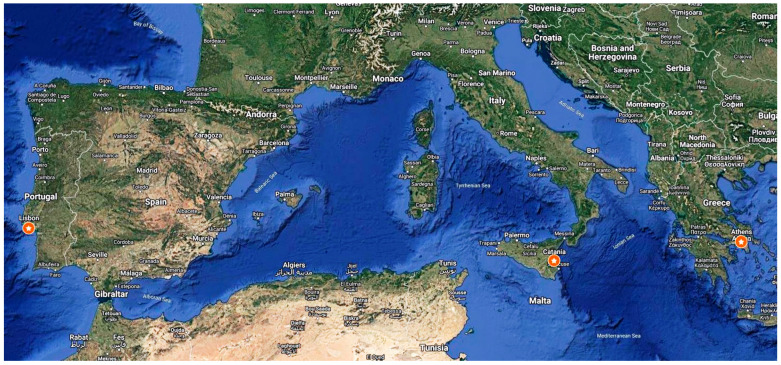
Map pointing out the three locations [Athens (Greece), Catania (Italy), and Caparica (Lisbon, Portugal)]. Map created by Google My Maps.

The 2-year experiments (2019 and 2020) were conducted in Greece, Italy, and Portugal (Figure 6), where five lignocellulosic crops were evaluated for their phytoremediation potential; namely, biomass sorghum *(Sorghum bicolor* L. Moench), switchgrass (*Panicum virgatum* L.), giant reed (*Arundo donax* L.), African fodder cane (*Saccharum spontaneum* L. spp. *aegyptiacum* Willd. Hackel), and miscanthus (*Miscanthus* × *giganteus*) as shown in Table 4.

The trials were conducted outdoors, and plants were maintained under well-watered conditions to ensure consistency. The main meteorological data were similar to the historical average at each site (Table 5). Also, the uncontaminated soil used for the pot trials was similar in the three countries where the trials were carried out.

### 4.1. Experimental Setup and Measurements

The main goal of our study was to simulate the relatively low levels of pollution commonly found in soil, aiming also to reflect the contamination levels observed in agricultural soils. To ensure that the experimental design was representative of real-farm conditions, the amount of heavy metals in soils was based on the maximum allowable concentrations of trace elements in agricultural soils, as outlined by the European Soil Directive (86/278/EEC) [60] as well as national legislations from Portugal, Italy, and Greece. Additionally, these levels align with the guidelines provided by Kabata-Pendias [47].

Specifically, the following max permissible concentrations were used as a basis for our calculations: cadmium (Cd): 3 mg kg^−1^, nickel (Ni): 75 mg kg^−1^, lead (Pb): 300 mg kg^−1^, and zinc (Zn): 300 mg kg^−1^.

The above concentrations were not applied directly in our study. Instead, they served as reference points. For the lowest treatment levels, we used 1.5 times the above-mentioned permissible concentrations, and for the highest treatment levels we applied twice the lowest treatment levels. This approach allowed us to simulate a range of contamination levels that closely approximate those commonly found in contaminated soils.

The research was carried out following the same protocols in pots with a capacity of 9.5 L and each pot was filled with 10 kg of soil (Table 6). Two months before sowing in spring, the heavy metals Cd, Ni, Pb, and Zn were applied to the soil as water solutions of nitrate salts [CdNO_3_·4H_2_O, purity > 98%, Merck KGaA Darmstadt, Germany; Pb(NO₃)₂, purity > 99%, Merck KGaA Darmstadt, Germany; Ni(NO_3_)_2_·6H_2_O, purity > 97%, Merck KGaA Darmstadt, Germany; Zn(NO_3_)_2_·6H_2_O, purity > 98%, Merck KGaA Darmstadt, Germany] in order to achieve low and high treatments. Three different quantities of each metal nitrate salt were used to achieve the following final concentrations in the soil:

(i) Contamination with Cd: 0, 4, 8 mg kg^−1^, which is referred to as Cd0 (control), Cd4 and Cd8 respectively.
(ii) Contamination with Pb: 0, 450, 900 mg kg^−1^, which is referred to as Pb0 (control), Pb450 and Pb900, respectively.
(iii) Contamination with Ni: 0, 110, 220 mg kg^−1^ which is referred to as Ni0 (control), Ni110 and Ni220, respectively.
(iv) Contamination with Zn: 0, 450, 900 mg kg^−1^, which is referred to as Zn0 (control), Zn450 and Zn900, respectively.

The experimental design was completely randomized, with three replicates for each treatment. In both years, after the germination of the seeded plants (i.e., sorghum and switchgrass), manual thinning was performed, leaving one plant per pot. Rhizomatous crops were transplanted through rhizome cuttings, with 2–3 main buds. The rhizome sizes and weights were as similar as possible. Giant reed and African fodder cane rhizomes were collected from the germplasm collection at the experimental farm of the University of Catania (37°24′ N, 15°03′ E, 10 m a.s.l.). Sorghum and switchgrass seeds were provided by the Center for Renewable Energy Sources (CRES, Athens, Greece), and miscanthus rhizomes were collected by the NOVA School of Science and Technology (NOVA FCT, Caparica, Portugal).

At the end of each experimental year, growth measurements were recorded per pot, namely the plant height, and fresh and dry above-ground weights. Finally, the heavy metal content in the above-ground plant was determined, as described below.

### 4.2. Plant Analysis

At harvest, the plants were thoroughly washed with tap water to remove soil particles, fresh weighed, then oven-dried (96 h at 60 °C) up to constant weight. Plant samples were ground using a cross-hammer beater mill and sieved with a 1 mm sieve. Determination of heavy metal content was carried out using atomic absorption spectroscopy (AAS). The digestion of plant samples was conducted in a microwave oven (Model speedwave Entry DAP-60, Berghof Products + Instruments GmbH, Eningen, Germany). Plant samples (0.3 g dry weight) were digested with 5 mL HNO_3_ and 2 mL H_2_O_2_. Then the samples were centrifuged for 10 min at 2500 rpm and filtered through a sterile mixed cellulose ester filter with 0.2 μm pore size. The sample volumes were adjusted to 25 mL using deionized water. Different heavy metals standard solutions such as cadmium had been prepared in order to create a curve of calibration using a cadmuim, zinc, lead, or nickel reference solution. All the solutions, including the extracted sample have been measured using a PerkinElmer atomic absorption spectrometer, AAnalyst 200.

### 4.3. Statistical Analysis and Phytoremediation Index

Statistical analysis was performed according to the experimental layout using R CRAN software version 4.4.0. The average values of the data for both years were used. As the data followed a normal distribution, one-way and two-way ANOVAs for each treatment were separately performed to compare the growth and ability of the five industrial crops as phytoremediators. The Duncan test was used to compare significantly different means at a confidence level of 95%.

The tolerance index (TI) was obtained by dividing the dry above-ground biomass of contaminated plants (g pot^−1^) by the dry above-ground biomass of control plants (g pot^−1^) [24,61].

(1) $$TI=\frac{dry\;aboveground\;biomass\;weight\;of\;contaminated\;plants,g\;{pot}^{-1}}{dry\;aboveground\;biomass\;wheight\;of\;control\;plants,g\;{pot}^{-1}}$$

The modified accumulation index (mAI) was calculated using the ratio between the metal accumulation in the contaminated plant (mg kg^−1^) and the heavy metal accumulation in the control plants (mg kg^−1^) [24,61].

(2) $$mAI=\frac{metal\;accumulation\;in\;the\;contaminated\;plants,mg\;{kg}^{-1}}{metal\;accumulation\;in\;the\;control\;plants,mg\;{kg}^{-1}}$$

In order to discriminate the differences in biomass yield among treatments, a PCA analysis of the heavy metals’ biomass concentrations and above-ground biomass was conducted. Furthermore, a multivariate analysis was carried out using a cluster analysis with Manhattan distance in order to identify similar behaviors within the crops tested.

## 5. Conclusions

This study assessed the performance of five lignocellulosic crops—switchgrass, sorghum, giant reed, African fodder cane, and miscanthus—grown in soils contaminated with Cd, Pb, Zn, and Ni. The results indicated diverse responses to heavy metal contamination. African fodder cane and miscanthus showed significant height reduction and decreased productivity under high Cd and Ni concentrations. Switchgrass seeds failed to germinate in highly Zn-contaminated soils, whereas sorghum, giant reed, and African fodder cane experienced notable height reductions. Miscanthus demonstrated resilience in terms of height, despite high Zn levels. Overall, all crops except miscanthus exhibited reduced biomass and metal uptake with increasing contamination, affecting their phytoremediation efficiency. However, switchgrass and sorghum exhibited higher tolerance and accumulation indices for Cd, Pb, and Zn, suggesting their potential for effective remediation. These findings highlight the varied strengths of these crops for phytoremediation and biomass production in contaminated soils, with specific crops being more suitable for different types of heavy metal contamination scenarios. The results highlight that the phytoremediation technique, particularly when non-hyperaccumulator plants are used, is a very slow process. However, the use of industrial crops could help reduce the remediation costs due to the feedstock obtained from the biomass, which could be used for energy production or other industrial purposes. Moreover, soil conditions and environmental constraints can affect the absorption of heavy metals. Despite this, investigated bioenergy grasses appear suitable for use in long-term remediation programs for polluted soils. Future research could focus on combining phytomanagement with chelators or microorganisms to enhance the extraction of heavy metals from the soil.

## Figures and Tables

**Table 1:** Two-way ANOVA of plants’ dry weights (g) for the five crops affected by the heavy metal levels. The crops and the levels of the heavy metal were used as fixed factors. Significance codes: *** *p* < 0.001, ** *p* < 0.01, n.s. *p* < 1.

Factors	Cadmium Pr (>F)	Lead Pr (>F)	Nickel Pr (>F)	Zinc Pr (>F)
Levels HM	***	***	***	***
Crops	***	***	***	***
Levels HM × Crops	n.s.	n.s.	n.s.	**

**Table 2:** Plants’ dry weights (g) of the five crops as affected by the heavy metal treatments. The values presented are the average values of two experimental years. Within crops, different lower-case letters indicate statistical significance (*p* < 0.05) comparing the control with the heavy metal levels.

Heavy Metal	Switchgrass	Sorghum	Giant Reed	African Fodder Cane	Miscanthus	Average
Control *	221.0 a	410.1 a	52.8 a	51.9 a	53.0 a	157.8
Cd4	259.5 a	336.3 ab	38.6 ab	40.1 a	43.4 a	143.6
Cd8	250.3 a	374.2 ab	36.7 ab	29.9 a	36.2 a	145.5
Pb450	216.8 a	384.2 ab	44.3 ab	49.4 a	50.2 a	149.0
Pb900	230.0 a	332.5 ab	18.6 cd	34.4 a	57.4 a	134.6
Ni110	230.0 a	471.2 a	35.2 bc	37.0 a	53.6 a	165.4
Ni220	220.7 a	237.9 ab	35.9 b	26.6 a	43.8 a	113.0
Zn450	209.5 a	345.8 ab	39.5 ab	43.6 a	43.2 a	136.3
Zn900	0.0 b	102.6 b	16.9 d	26.1 a	40.9 a	37.3

* Natural soil without any added contaminants.

**Table 3:** Heavy metal concentrations (mg kg^−1^ of dry matter) in the aerial biomass of plants for the years 2019 and 2020. Within crops and heavy metals, different lower-case letters indicate statistical significance (*p* < 0.05) comparing the control with the different levels of heavy metals tested.

	Switchgrass (mg kg^−1^)	Sorghum (mg kg^−1^)	African Fodder Cane (mg kg^−1^)	Giant Reed (mg kg^−1^)	Miscanthus (mg kg^−1^)
Control	5.3 b	8.9 a	2.0 c	2.1 b	1.1 b
Cd4	12.6 a	14.8 a	13.6 b	15.8 a	1.4 b
Cd8	13.4 a	15.8 a	17.5 a	19.9 a	7.4 a
Avg	10.43	13.17	11.03	12.60	3.30
Control	9.6 c	21.0 b	4.3 b	5.6 b	14.5 a
Pb450	24.9 b	25.1 b	12.9 ab	14.7 ab	18.9 a
Pb900	39.5 a	55.5 a	16.4 a	24.4 a	18.2 a
Avg	24.67	33.87	11.20	14.90	17.20
Control	8.7 c	1.2 b	9.3 b	10.8 b	27.3 b
Ni110	22.5 b	6.7 ab	28.8 a	24.2 a	138.8 b
Ni220	32.7 a	10.2 a	22.9 a	29.8 a	296.8 a
Avg	21.30	6.03	20.33	21.60	154.30
Control	3.0 b	10.0 b	68.7 c	76.9 b	21.7 c
Zn450	105.1 a	104.0 a	116.7 b	92.9 b	77.1 b
Zn900	-	100.9 a	168.9 a	323.4 a	140.8 a
Avg	54.05	71.63	118.10	164.40	79.87

**Table 4:** Crop allocation (+) and experimental sites per country.

	Greece, Athens, AUA 37°59′ N, 23°42′ Ε, 33 m a.s.l.	Italy, Catania, UNICT 37°31′ N, 15°04′ E, 75 m a.s.l.	Portugal, Caparica, NOVA FCT 38°40′ N, 9°12′ W, 99 m a.s.l.
Sorghum	+		
Switchgrass	+		
Giant reed		+	
African fodder cane		+	
Miscanthus			+

**Table 5:** Main meteorological data (monthly mean temperature and rainfall) per location.

	GREECE				ITALY				PORTUGAL			
	2019		2020		2019		2020		2019		2020	
	Temp (°C)	Precip (mm)	Temp (°C)	Precip (mm)	Temp (°C)	Precip (mm)	Temp (°C)	Precip (mm)	Temp (°C)	Precip (mm)	Temp (°C)	Precip (mm)
January	9.7	125.0	9.2	16.4	9.2	38.0	9.78	14.8	10.4	34.2	11.1	64.9
February	10.1	59.2	11.3	12.0	11.1	67.0	11.4	3.4	12.0	35.0	13.1	7.5
March	13.6	26.8	13.3	48.2	13.5	6.8	12.0	214.2	14.5	24.5	14.2	20.1
April	15.4	115.0	15.3	18.6	14.8	25.6	14.9	11.2	14.9	48.2	15.5	111.4
May	19.9	2.2	21.2	32.2	16.6	18.8	19.2	0.6	20.3	7.9	20.4	73.1
June	26.8	2.6	24.8	16.8	23.8	1.8	22.1	5.6	19.5	7.9	20.8	1.8
July	28.2	1.0	28.8	0.0	26.6	11.0	25.6	34.8	22.8	0.6	24.6	2.0
August	29.3	0.0	28.5	17.4	26.8	14.4	27.1	0.4	23.8	0.7	23.9	0.9
September	24.8	4.8	26.0	5.0	23.8	51.4	23.8	162.2	22.1	24.9	22.0	27.3
October	21.3	23.4	20.8	35.2	20.6	170.4	18.5	11.4	18.0	55.8	18.1	118
November	17.7	120.4	14.9	4.6	15.0	153.2	15.5	215.4	14.3	76.4	15.4	55.9
December	12.2	90.6	13.6	119.8	12.8	14.6	12.1	81.8	12.5	130.5	11.3	76.6

**Table 6:** Main soil characteristics per country.

	Greece	Italy	Portugal
Physical characteristics			
Clay (%)	20.6	3.0	47.1
Silt (%)	4.0	4.1	29.7
Sand (%)	75.4	92.9	23.2
Texture	Sandy loam	Sandy	Clay
Conductivity (μS/cm)	28.9	34.2	100.0
Chemical characteristics			
pH	7.8	7.4	7.7
Organic matter (g 100^−1^ g DW)	1.17	0.86	0.31
CEC (cmol(+)kg^−1^, DW)	8.8	6.3	9.3
Total nitrogen (g N kg^−1^, DW)	0.22	0.06	0.29
Available phosphorus (mg P kg^−1^, DW)	16.9	10.0	25.5
Total potassium (g K kg^−1^, DW)	3.3	4.5	2.1
Total calcium (g Ca kg^−1^, DW)	56.0	79.8	43.0
Total sodium (g Na kg^−1^, DW)	2.10	0.31	1.50
Total magnesium (g Mg kg^−1^, DW)	1.23	0.68	1.99
Total Cd (mg kg^−1^)	<d.l.	<d.l.	<d.l.
Total Pb (mg kg^−1^)	34.06 ± 2.89	17.45 ± 3.96	23 ± 3
Total Zn (mg kg^−1^)	60.34 ± 6.26	36.89 ± 5.50	68 ± 4
Total Ni (mg kg^−1^)	63.59 ± 4.88	20.92 ± 4.76	8.44 ± 0.11

## Data Availability

The raw data supporting the conclusions of this article will be made available by the authors on request.
